# Next-Generation Sequencing and Genome Editing in Plant Virology

**DOI:** 10.3389/fmicb.2016.01325

**Published:** 2016-08-26

**Authors:** Ahmed Hadidi, Ricardo Flores, Thierry Candresse, Marina Barba

**Affiliations:** ^1^United States Department of Agriculture – Agricultural Research ServiceBeltsville, MD, USA; ^2^Instituto de Biología Molecular y Celular de Plantas, Universidad Politécnica de Valencia–Consejo Superior de Investigaciones CientíficasValencia, Spain; ^3^UMR 1332 Biologie du Fruit et Pathologie, Institut National de la Recherche Agronomique, Université de BordeauxBordeaux, France; ^4^Consiglio per la Ricerca in Agricoltura e l’analisi dell’Economia Agraria, Centro di Ricerca per la Patologia VegetaleRome, Italy

**Keywords:** next-generation sequencing, plant virology, plant viruses, viroids, resistance to plant viruses by CRISPR-Cas9

## Abstract

Next-generation sequencing (NGS) has been applied to plant virology since 2009. NGS provides highly efficient, rapid, low cost DNA, or RNA high-throughput sequencing of the genomes of plant viruses and viroids and of the specific small RNAs generated during the infection process. These small RNAs, which cover frequently the whole genome of the infectious agent, are 21–24 nt long and are known as vsRNAs for viruses and vd-sRNAs for viroids. NGS has been used in a number of studies in plant virology including, but not limited to, discovery of novel viruses and viroids as well as detection and identification of those pathogens already known, analysis of genome diversity and evolution, and study of pathogen epidemiology. The genome engineering editing method, clustered regularly interspaced short palindromic repeats (CRISPR)-Cas9 system has been successfully used recently to engineer resistance to DNA geminiviruses (family, *Geminiviridae)* by targeting different viral genome sequences in infected *Nicotiana benthamiana* or *Arabidopsis* plants. The DNA viruses targeted include tomato yellow leaf curl virus and merremia mosaic virus (begomovirus); beet curly top virus and beet severe curly top virus (curtovirus); and bean yellow dwarf virus (mastrevirus). The technique has also been used against the RNA viruses zucchini yellow mosaic virus, papaya ringspot virus and turnip mosaic virus (potyvirus) and cucumber vein yellowing virus (ipomovirus, family, *Potyviridae)* by targeting the translation initiation genes eIF4E in cucumber or *Arabidopsis* plants. From these recent advances of major importance, it is expected that NGS and CRISPR-Cas technologies will play a significant role in the very near future in advancing the field of plant virology and connecting it with other related fields of biology.

## Introduction

The field of virology was born in the late 1890s when it was found that the tobacco mosaic disease is caused by a novel form of infectious agent named “ultravirus” and referred to as ‘contagium vivum fluidum’ (soluble living germ or contagious living fluid; Hadidi and Barba, 2012). The virus, later named tobacco mosaic virus (TMV), was the first to be described and become an iconic one, especially in the first half of the 20th century. TMV was purified and crystallized by Wendell M. Stanley in 1935, who also described its structure, and Heinz Fraenkel-Conrat in 1950s was the first to show that its replication is directed by genetic information encoded within its RNA core (Hadidi and Barba, 2012), thus starting molecular virology. Molecular virology was also instrumental in the discovery, by Theodor O. Diener, of a novel class of infectious small RNA in 1971 as the causal agent of potato spindle tuber disease (Diener, 1971a,b), which he later named potato spindle tuber viroid (PSTVd; Diener, 1972). Viroids are very small circular, non-coding RNAs (Sänger et al., 1976; Owens et al., 1977). Unlike virus particles (virions), which are made of protein coats encapsidating nucleic acid genomes, viroids are naked single-stranded (ss) RNAs without protein coats, 247–401 nt in length and with a high degree of internal base pairing; they are the smallest known infectious agents (Hadidi et al., 2003). The 2012 Report of the International Committee for the Taxonomy of Viruses (ICTV) lists about 900 plant virus species (King et al., 2012). The current number of viroid species is 32 (Di Serio et al., 2014). Viruses and viroids infect vegetable, field, ornamental, and/or tree crops as well as wild plant species, in which they may induce significant effects on plant health such as a decrease of desired quality or loss of yield, causing a reduction in income for farmers, other crop producers, and distributors, and higher prices for consumers. Moreover, increased international movement, globalization of trade, propagation material, and seed supply systems all influence the introduction and proliferation of virus and viroid diseases across countries. Crop yield reductions attributed to specific virus or viroid infections in specific crops may vary from less than 10% to more than 80–90% for viruses (Waterworth and Hadidi, 1998; Hadidi et al., 2011; Barba and Hadidi, 2015) and from 17% to close to 100% for viroids (Singh et al., 1971; Zelazny et al., 1982; Hadidi et al., 2003). Some viruses and viroids can be largely latent (symptomsless) in some of their infected hosts (Hadidi et al., 1998, 2003, 2011). These latent agents, however, may be pathogenic in other hosts and their infections may result in yield reduction and general weakness of plants (Hadidi et al., 1998, 2003, 2011; Hadidi and Barba, 2012; Barba et al., 2015).

The very large variability among plant viruses and among viroids complicates their discovery, detection, quarantine, and certification as well as their etiological studies by standard methods, which could be overcome or made easier by next-generation sequencing (NGS) technology. Similarly, despite progress in understanding virus or viroid–plant interactions, obtaining plants resistant to these pathogens by conventional breeding or transgenic strategies are often complicated and faces the problem of resistance durability. Virus or viroid genome and/or plant gene editing is expected to play a significant role in developing transgene-free plants resistant to viruses or viroids. Advances in NGS capabilities and the rapid development and widespread adoption of a simple, inexpensive, easy to use and very effective genome engineering editing method known as “clustered regularly interspaced short palindromic repeats and their associated Cas proteins (CRISPR-Cas)” are revolutionizing the fields of genetics, genomics, molecular biology and others. In this article we discuss the progress made in plant virology during the last 7 years by NGS and very recently by CRISPR-Cas systems.

## NGS in Plant Virology

Next-generation sequencing, combined with informatics for *de novo* discovery and assembly of plant virus or viroid genome reads, has been used since 2009, first in discovering novel DNA and RNA viruses (Adams et al., 2009; Al Rwahnih et al., 2009; Kreuze et al., 2009), and in detecting and identifying RNA viruses (Al Rwahnih et al., 2009; Donaire et al., 2009; Kreuze et al., 2009), as well as viroids (Al Rwahnih et al., 2009). Moreover, it was used in the same year for sequencing viroid-derived small RNAs (vd-sRNAs) to study the role of RNA silencing in plant–viroid interactions, particularly for nuclear-replicating viroids (Navarro et al., 2009), and to investigate the genesis and possible pathogenesis of vd-sRNAs from a chloroplast-replicating viroid (Di Serio et al., 2009). Subsequent data support a role of vd-sRNAs in viroid pathogenesis (Navarro et al., 2012a; Adkar-Purushothama et al., 2015; Avina-Padilla et al., 2015), as previously shown for an sRNA derived from the satellite RNA Y of cucumber mosaic virus (CMV; Smith et al., 2011; Shimura et al., 2011). vd-sRNAs and virus small RNAs (vsRNAs), 21–24 nt in length, are generated in host plants by their silencing machinery in response to infection by these foreign replicons. RNA silencing is a cell surveillance system that recognizes double-stranded (ds) RNA and ssRNA with a compact secondary structure, and specifically inactivates viroids and RNA viruses (by post-transcriptional gene silencing) as well as DNA viruses (by transcriptional and/or post-transcriptional gene silencing), using small interfering RNAs as a guide (for review see: Barba and Hadidi, 2009; Sano et al., 2010; Hammann and Steger, 2012; Navarro et al., 2012b; Wang et al., 2012; Flores et al., 2015; Zhang et al., 2015).

Next-generation sequencing of vsRNAs or vd-sRNAs has also been used in various studies on viruses or viroids, which include, but are not limited to, their characterization, profiling, distribution, accumulation, biogenesis, as well as their use in extending the known pathogen host range, and in virus strain differentiation, systemic movement, virus or viroid-host interaction, viroid evolution, and pathogenesis, mutation, mRNA targeting, and others (**Tables 1** and **2**).

**Table 1:** Utilization of NGS in various studies of plant virus small RNAs.

Virus small RNAs (vsRNAs)	Reference
vsRNAs of nine different viruses in four different hosts extended the knowledge of distribution and composition of these RNAs in virus-infected plants and contributed to better understanding of vsRNAs biogenesis	Donaire et al., 2009
Identification of two novel badnaviruses (dsDNA) and one novel mastrevirus (ssDNA) in sweet potato plants and detection of the often symptomless sweet potato feathery mottle virus and the phloem-limited sweet potato chlorotic stunt virus in the same plants	Kreuze et al., 2009
vsRNA of tobacco mosaic virus mediate virus-host interactions which may contribute to viral pathogenicity and host specificity	Qi et al., 2009
vsRNAs profiles of cymbidium ringspot virus were obtained. These RNAs primarily derived from the positive strand of the virus, accumulated with different frequency, had a 5′monophosphate, and were not perfect duplexes	Szittya et al., 2010
Characterization of vsRNAs from the four genome RNAs of rice stripe virus in infected rice plants	Yan et al., 2010
Profiling vsRNAs of bamboo mosaic virus and its associated satellite RNAs	Lin et al., 2010
vsRNAs of four virus genera (*Foveavirus*, *Maculavirus*, *Marafivirus* and *Nepovirus*) originate from both genomic and antigenomic strands. In addition, most vsRNAs of members of the genus *Tymovirus* are derived from the antigenomic virus strand	Pantaleo et al., 2010
Extending the host range of cereal yellow dwarf virus (genus *Luteovirus*) to wild cocksfoot grass	Pallett et al., 2010
Characterization of vsRNAs from tomato yellow leaf curl virus and its associated beta satellite DNA in infected tomato and *Nicotiana benthamiana* plants	Yang et al., 2011
Characterization of vsRNAs of cotton leafroll dwarf virus in infected cotton plants	Silva et al., 2011
Identification of a novel badnavirus in grapevine. It is the first DNA virus discovered in this host	Zhang et al., 2011
Detection of tomato spotted wilt virus in tomato before symptoms appeared at levels too low for conventional detection methods. Analysis of the virus quasispecies; identification of a tospovirus and a squash-infecting geminivirus	Hagen et al., 2011
vsRNAs sequencing reconstructed the full genome of the T318A Spanish citrus tristeza virus isolate that infects sweet and sour orange as well as Mexican lime. vsRNAs map preferentially at the 3′-terminal region of the genomic RNA. Virus infection affect the host sRNA profiles	Ruiz-Ruiz et al., 2011
Characterization of vsRNAs and transcriptome profiling of *Arabidopsis* plants infected by oil seed rape mosaic virus (genus *Tobamovirus*).	Hu et al., 2011
vsRNAs of both sense and antisense polarities without gaps densely cover the circular genome of DNA viruses, thus enabling *de novo* reconstruction of the complete DNA virus from vsRNAs	Blevins et al., 2011; Aregger et al., 2012; Rajeswaran et al., 2012
vsRNAs of rice stripe virus were shown to be generated preferentially in different plant hosts and they were identified in the viruleferous vector small brown leafhopper	Xu et al., 2012
Identification and differentiation of two strains of pepino mosaic virus and complete genome sequences of a novel potyvirus named tomato necrotic stunt virus in tomato. Identification of gene expression changes associated with disease	Li et al., 2012
Detection of sweet potato members of different genera *(Potyvirus, Crinivirus, Begomovirus*) in sweet potato. vsRNAs NGS analysis is a reliable and sensitive method for virus detection in infected crops	Kashif et al., 2012
Identification of a novel member of the genus *Mandarivirus* in citrus	Loconsole et al., 2012a
Identification of a novel DNA virus member of the family *Geminiviridae* in citrus	Loconsole et al., 2012b
Detection of four apple viruses and two apricot viruses associated with apple green crinkle, a disease of undermined etiology	Yoshikawa et al., 2012
Identification of a novel members of the genus *Tricovirus*, grapevine pinot gris virus, in grapevine cv. Pinot gris. Detection of grapevine rupestris stem-pitting associated virus, grapevine rupestris vein feathering virus and grapevine Syrah virus 1	Giampetruzzi et al., 2012
Characterization of vsRNA associated with grapevine leafroll disease	Alabi et al., 2012
vsRNAs sequencing reconstructed the full genome of the Mexican tomato mottle mosaic virus, genus *Tobamovirus*, that infects tomato	Li et al., 2013
Identification of a novel member of the genus *Enarnovirus*, citrus vein enation virus, in Etrog citron plants	Vives et al., 2013
Identification of a novel member of the genus *Cilevirus*, citrus leprosies virus cytoplasmic type 2, in citrus	Roy et al., 2013
vsRNAs of tomato spotted wilt virus accumulate at different amounts in different hosts such as tomato and *Nicotiana benthamiana*	Mitter et al., 2013
vsRNAs profiles of potato virus Y strains O, N, and NTN were different in the same host which indicated they interact differently. vsRNAs were derived from every position in the genome and certain hot spots were identified for each strain	Naveed et al., 2014
vsiRNAs of potato virus X were successfully differentiated according to their strains	Kutnjak et al., 2014
vsRNAs and vd-sRNAs allowed *de novo* reconstruction of DNA and RNA viruses or viroids and their variants. vsiRNAs could be used for diagnosis of known and emerging virus or viroid diseases and for assessing rapid generation of biologically active clones	Seguin et al., 2014
vsRNAs profiles of apple stem grooving virus latent infection in apple seedlings showed an increase toward the 3′end of the virus genome. The involvement of tRNA-derived sRNAs in plant–virus interaction was demonstrated	Visser et al., 2014
vsRNAs of zucchini mosaic virus were used to study the systemic movement of the virus within the inoculated leaf of *Cucurbita pepo*. The number of virus variants increases with the distance from the inoculation site and the variant mutations resulted in significant conformation in the cylindrical inclusion protein	Dunham et al., 2014
Identification of a novel geminivirus, genus *Mastrevirus*, named sugarcane white streak virus in post quarantine sugarcane plant material. The accumulating vsRNAs are strongly influenced by secondary structures within both the viral genomic single-stranded DNA and its mRNA transcripts	Candresse et al., 2014
Identification and molecular characterization of a novel monopartite geminivirus associated with mulberry mosaic dwarf disease	Ma et al., 2015
Identification and characterization of a novel geminivirus with a monopartite genome infecting apple trees	Liang et al., 2015
Identification of a new genotype of squash mosaic virus in squash grown in Spain	Li et al., 2015
Extending the host range of tomato mottle mosaic virus, genus *Tobamovirus*, to chickpea *(Cicer arietinum)* with red seeds and its geographical distribution to Europe	Pirovano et al., 2015
First comparative analysis among the vsRNAs of source, sink and conductive (phloem) tissues in two different plant–virus pathosystems. Melon and cucumber plants were infected with melon necrotic spot virus and prunus necrotic ringspot, respectively, two viruses differing in genome organization and replication strategy. The vsRNA profile remains constant in phloem but not in the other tissues. vsRNAs share the same size distribution in all analyzed tissues. Both viruses were able to modulate the host sRNA profile.	Herranz et al., 2015
Identification and molecular characterization of a novel closterovirus named rose leaf rosette-associated virus	He et al., 2015

**Table 2:** Utilization of NGS in various studies of viroid small RNAs.

Viroid small RNAs (vd-sRNAs)	Reference
vd-sRNAs of peach latent mosaic viroid were used to study the viroid evolution and pathogenesis	Di Serio et al., 2009; Navarro et al., 2012a
Gaining further insights into the genesis and role of vd-RNAs of hop stunt viroid and grapevine yellow speckle viroid 1 in plant–viroid interaction	Navarro et al., 2009
vd-sRNAs from PSTVd-infected wild-type and RDR6i *Nicotiana benthamiana* plants accumulate to levels paralleling their genomic RNA, display similar patterns with prevailing 22- or 21-nt plus-strand species, and adopt strand-specific hot spot profiles	Di Serio et al., 2010
The pathway involved in the biogenesis of vd-sRNAs of hop stunt viroid was studied and revealed	Martinez et al., 2010
Characterization of vd-sRNA of hop stunt viroid, grapevine yellow speckle 1 and grapevine yellow speckle 2 viroids in grapevine	Alabi et al., 2012
vd-sRNAs of peach latent mosaic viroid (PLMVd) were characterized. Similarly to host microRNAs (miRNAs), two PLMVd vd-sRNA derived from the pathogenic determinant of an extreme albinism direct cleavage of a specific host mRNA, strongly suggesting their involvement in symptom expression	Navarro et al., 2012a
Based on the observation that viroid-infected plants generate vd-sRNAs 21–24 nt, an approach was developed to utilize several bioinformatic tools for identifying novel and known viroids and viroid-like circular RNAs in sRNA libraries	Wu et al. (2012)
vd-sRNAs derived from potato spindle tuber viroid variants inducing different symptoms, may aim at multiple and different host mRNA targets	Wang et al., 2011; Piernikarczyk et al., 2013
Computational algorithms as bioinformatic tools were utilized to identify circular RNAs of viroid or satellite sRNAs	Zhang et al., 2014
Extending the host range of apple dimple fruit viroid to fig	Chiumenti et al., 2014
Inoculation with a single variant of peach latent mosaic viroid generates a highly heterogeneous progeny within a single infected peach seedling. The most distant variants displayed a 17% variation level when compared to the parent sequence	Glouzon et al., 2014
vd-sRNAs of potato spindle tuber viroid (PSTVd) and effects of artificial miRNA derived from PSTVd-mild or -severe infected plants were analyzed. Differences in the distribution of vdsRNAs hot spots were observed. Data suggest involvement of vd-sRNAs in symptom expression	Adkar-Purushothama et al., 2015; Avina-Padilla et al., 2015
Extending the host range of hop stunt viroid to chickpea	Pirovano et al., 2015

Next-generation sequencing can in a single experiment determine the sequence of hundreds of thousands to millions of vsRNA or vd-sRNA, which can be re-assembled to obtain the genomic sequence of the virus or viroid genome(s) of interest but can also be compared to the host genome in an effort to identify genes that may be down-regulated upon infection as a consequence of their local homology with the infecting virus or viroid. In parallel, the identification in otherwise healthy plants of sRNAs with homology to viral satellite RNAs has provided recently a tentative scenario for their evolution from the host plant genome, offering a possible solution to the long lasting conundrum of the origin of these infectious agents (Zahid et al., 2015; Wang and Smith, 2016).

### Discovery and Diagnostics of Plant Viruses and Viroids by NGS

Next-generation sequencing has significantly increased the number of novel plant viruses discovered and characterized both in host plants and in insect vectors. More than 100 novel DNA and RNA plant viruses from different genera and families have been reported in the recent years (Hadidi and Barba, 2012; Barba et al., 2014; Ho and Tzanetakis, 2014; Barba and Hadidi, 2015; Roossinck et al., 2015; Wu et al., 2015). Only two novel viroids, however, were discovered: persimmon viroid 2 (Ito et al., 2013) and grapevine latent viroid (Zhang et al., 2014). This trend has been observed in crop plants but also, to a very large extent, in wild plant species through the use of NGS in metagenomic approaches (Stobbe and Roossinck, 2014; Roossinck, 2015; Roossinck et al., 2015). Likewise, the sequences of many novel virus and viroid strains have been reported. In addition to discovering novel viruses, the complete nucleotide sequences of many known viruses were determined by NGS for complete virus characterization and/or virus identification in known and new hosts or for other reasons. For example, NGS has shown that the artichoke latent virus (ArLV) is a member of the genus *Macluravirus*, family *Potyviridae*, and that ranunculus latent virus should be considered as a strain of ArLV but not a distinct species (Minutillo et al., 2015); potato virus Y and potato virus S have been identified in Maori potato (*Solanum tuberosum*) and turnip mosaic virus in rengarenga (*Arthropodium cirratum*), which is a new host (Blouin et al., 2016). NGS analyses do not generally provide the final word on a new virus or viroid. The genome sequence generally has to be finalized using PCR-based approaches and Sanger sequencing and, as a general rule, the existence of the new virus or viroid should always be sought using an alternative technique. In addition, NGS librarary prepation methods with minimal bias should be used in order to obtain accurate and easy to interpret data (Van Dijk et al., 2014).

Another area where NGS has proven very valuable is in the detection of isolates, strains, or variants of known viruses that escape existing detection procedures and, particularly, PCR assays. The data obtained may afford a better knowledge of the polyvalence or specificity of existing assays and, if needed, facilitate the design of new detection primers of broader specificity for improving the classical detection assays. For example, the NGS discovery of a non-detectable isolate of plum bark necrotic and stem pitting associated virus led Marais et al. (2014) to develop a new PCR assay of broader specificity.

NGS has provided a very powerful alternative for detection and identification of plant viruses and viroids without *a priori* knowledge of pathogen sequence as required for PCR-based detection and identification methods. For this reason, NGS has become a universal approach for accurate detection and identification of many novel and known plant viruses. Viroids are also accurately and easily detected and identified by NGS (**Table 3**). Thus, NGS has the potential to be used as a primary diagnostic tool for plant viruses and viroids as the cost of sequencing platforms has become more competitive and affordable. Currently, the cost of NGS-based diagnostics is still high as compared to that of a PCR or serological assay, so that the technique is limited to situations where exhaustiveness is critical, such as quarantine or when trying to identify a causal agent, or to situations involving high value added samples, such as nuclear stock mother plants used for production of certified planting materials. It should be stressed that nucleic acids purifications and sequencing bank preparation protocols may have to be optimized and fine-tuned/adapted to particular plant species that may contain inhibitory substances that may otherwise interfere with the sensitivity of the detection procedure.

**Table 3:** Detection and identification of viroids by NGS.

Viroid	Target	Reference
Potato spindle tuber viroid	sRNAs	Diermann et al., 2010; Di Serio et al., 2010; Wang et al., 2011; Li et al., 2012; Adkar-Purushothama et al., 2015
Citrus exocortis viroid	Total RNA	Poojari et al., 2013
Apple dimple fruit viroid	sRNAs	Chiumenti et al., 2014
Peach latent mosaic viroid	sRNAs	Di Serio et al., 2009; Bolduc et al., 2010; Glouzon et al., 2014
Hop stunt viroid	sRNAs	Navarro et al., 2009; Martinez et al., 2010; Alabi et al., 2012; Giampetruzzi et al., 2012; Seguin et al., 2014; Pirovano et al., 2015
	Total RNA	Poojari et al., 2013
Citrus bark cracking viroid	Total RNA, sRNAs	Jakse et al., 2015
Grapevine yellow speckle viroid 1	sRNAs	Navarro et al., 2009; Martinez et al., 2010; Giampetruzzi et al., 2012; Seguin et al., 2014
	Total RNA	Poojari et al., 2013; Jo et al., 2015
Grapevine yellow speckle viroid 2	sRNAs	Alabi et al., 2012
Grapevine latent viroid	Total RNA	Zhang et al., 2014
Persimmon viroid 2	dsRNA	Ito et al., 2013
Pathogenic circular RNAs	sRNA	Wu et al., 2012

The volume and diversity of international movements of plant materials, including exchanges of germplasm or newly bred cultivars, have increased considerably during the last few decades, which have also created additional pathways for the introduction of plant viruses and viroids in new areas or for the emergence of novel virus or virus-like agents. Preventive measures for blocking the introduction and spread of these pathogens include the implementations of plant quarantine (phytosanitary regulation) and certification programs. These programs are tools that can be used to protect or improve the health status of cultivated plants (Foster and Hadidi, 1998; Barba, 1998; Barba et al., 2003; Singh et al., 2003; Reed and Foster, 2011; Roy, 2011). NGS has played a significant role in revealing new viruses and viroids of quarantine and/or certification importance. For example, imported sugarcane plants in quarantine in Montpellier, France, were found infected with a novel geminivirus, tentatively named sugarcane white streak virus, which had escaped detection by standard detection tests (Candresse et al., 2014). Similarly, NGS allowed the identification of a novel luteovirus in imported nectarine trees in the US (Bag et al., 2015; Villamor et al., 2016) and of a novel marafivirus (Villamor et al., 2016), suggesting that this technology could/should be adopted as a post-entry quarantine measure (see below). Also, very recently, NGS revealed that the causal agent of severe stunting and death of hop plants in Slovenia is citrus bark cracking viroid (CBCVd; Jakse et al., 2015), a pathogen reported previously in citrus plants wherein it induces minor damage (Duran-Vila and Semancik, 2003). Subsequently, CBCVd has been added to the certification list of hop planting material in Slovenia; in addition, the European and Mediterranean Plant Protection Organization (EPPO) has included the viroid to “The EPPO Alert List” (Jakse et al., 2015), so member countries and other countries may include it in their certification or quarantine programs or become aware of potential problems.

Recently, the US Department of Agriculture, Animal and Plant Inspection Service (APHIS), Plant Protection and Quarantine (PPQ) formed an internal working group to discuss the application of NGS to PPQ policy and operations. There are a number of challenges, however, that have to be addressed on the use of NGS in quarantine before a regulatory policy can be implemented. These include standardization of testing methods, interpretation of test results, biology of the discovered new pathogen, constructing a reliable database of whole genome sequence of pathogens of quarantine importance and others (E. V. Podleckis, personal communication 2016; M. K. Nakhla, personal communication 2016). It is expected that these challenges will be soon resolved due to advances in NGS capabilities and the rapid adaptation of this technology in plant pathogen diagnostics. NGS can become instrumental in releasing plants in quarantine and certification programs at a faster rate than current strategies while improving our ability to prevent the introduction of foreign plant viruses and/or viroids into new countries. Thus, NGS has the potential to be utilized in plant quarantine and certification programs standard assays in North America and Europe in the near future as, when compared with routine conventional assays, it could reduce significantly the number of non-detected viruses and viroids.

### Relationship of DNA Green Algal Viruses and ssRNA Plant Viruses to Human Disorders/Diseases as Revealed by NGS

While no plant virus has been shown so far to be harmful to humans, the use of NGS approaches has yielded recently a few tantalizing results that may in time question this long held vision. Sequences homologous to the DNA of the green algal virus acanthocystis turfacea chlorella virus 1 (ATCV 1) were detected and identified in human oropharyngeal samples of healthy normal adults without any physical or mental disorder/illness (Yolken et al., 2014). Mice inoculated with ATCV 1, however, developed memory loss and other symptoms indicating a general decrease performance in several cognitive domains. On the other hand, DNA viruses that infect the green alga *Phaeodactylum tricornutum* have been associated with vaginitis (Stepanova et al., 2011). Women with this disease swam in the Black Sea two to three months before the symptoms appeared. Very recently, it was shown by NGS that the green alga DNA virus TsV-N1 that infects *Tetraselmis striata* has two genes with closest similarity to genes in parasites of the human urogenital system, *Trichomonas vaginalis* and *Candida albicans* (Pagarete et al., 2015).

Similarly, the observation that the RNA viral community of the human feces is dominated by plant viruses (Zhang et al., 2006) came as a surprise. It prompted further efforts that led to a report that the ssRNA pepper mild mottle virus (PMMV) was highly represented in a human population, that anti PMMV IgM antibodies could be detected in some persons, and that some relationship between PMMV detection and some clinical symptoms might exist (Colson et al., 2010). These findings might be also correlated with a report showing that the negative ssRNA tomato spotted wilt virus (TSWV) is able to replicate in human cell lines expressing the viral “polymerase-bound host factor” (de Medeiros et al., 2005).

More insights into the role of PMMV, TSWV, algal viruses and other higher plant and algal viruses in human diseases may be revealed by metagenomic studies using NGS of human gut viromes and other organs of patients (Balique et al., 2015). Thus, the relationship between these viruses and human disorders/diseases may need serious re-evaluation.

### Possible Sequencing of Old/Ancient Viruses and Viroids by NGS

NGS technology advances, plus new sample preparation techniques, have allowed researchers to sequence complete ancient genomes from modern human ancestors and archaic humans (Gibson, 2015). NGS has also revealed the sequence of the ancient DNA of the 19th century late blight oomycetes pathogen, *Phytophthora infestans*, which caused the Irish potato famine of 1845–1847 (Martin et al., 2013). The high quality of the pathogen DNA in the 166–168 years old herbarium material suggests that DNA and RNA plant viruses as well as viroids in dried plant samples in herbaria, museums, or other places world-wide, could be used for studies of past epidemics and/or evolution of these pathogens by NGS. Studies using RT-PCR identified peach latent mosaic viroid in 50-year-old herbarium-preserved peach leaves showing symptoms of peach calico disease (Guy, 2013), and apple scar skin viroid in a 10-year-old air dried apple tree twig with no disease symptoms (A. Hadidi, unpuplished data). Plant viruses were also reported in 50- to 100-year-old herbarium samples using different traditional detection methods (for references, see Guy, 2013). In what may be the most remarkable result to date, Smith et al. (2014) were able to assemble the complete genome of a barley stripe mosaic virus (BSMV) isolate from small RNA sequences from barley grains that were approximately 750 years old. Interestingly, the sequence obtained does not fit well the phylogenetic reconstruction of the evolutionary timeline for BSMV, questioning the previously reconstructed history of BSMV and the hypothesis of a recent origin of the virus. Similarly, aged or older plant and soil samples could also be analyzed by NGS for plant viruses and viroids, as illustrated by the recent discovery of viral genomes in 700-years old caribou feces from a subarctic ice patch (Ng et al., 2014) and of a giant DNA virus named pithovirus sibericum in a 30,000 years old Siberian permafrost sample (Legendre et al., 2014). Such analyses would potentially allow to gain knowledge on the evolutionary history of plant viruses and viroids over the past few millennia, a time period hypothesized to have seen the emergence of several very important viral genera, as for example the evolutionary radiation of the potyviruses (Gibbs et al., 2008), the most important and numerous plant virus genus.

## Genome Editing Using the CRISPR-Cas9 System in Plant Virology

CRISPR associated Cas proteins (CRISPR-Cas) systems act as archaea and bacteria immune systems against invading foreign DNAs (Mojica et al., 2005; Barrangour et al., 2007; Makarova et al., 2011; Sorek et al., 2013). The Cas proteins, such as Cas9, are RNA-directed endonucleases able to recognize and cleave nucleic acids on the basis of sequence complementarities (Brouns et al., 2008; Bhaya et al., 2011; Gasiunas et al., 2012; Jinek et al., 2012; Westra et al., 2012; Sorek et al., 2013) and to modify the targeted sequences (Hsu et al., 2014). Cas9 can be targeted to specific DNA genomic sequences by engineering separately an encoded small guide RNA (sgRNA) with which it forms a complex (Doudna and Charpentier, 2014). Thus, only a short RNA sequence must therefore be synthesized to confer recognition of a new target. Recently, an alternative to the Cas9 enzyme, Cpf1, has been reported (Zetsche et al., 2015), which is smaller in size and makes genome editing easier and more precise.

RNA-guided cleavage paired with donor-guided repair allows easy introduction of any desired modification in a living cell. The possibility of re-directing the dsDNA targeting capability of CRISPR-Cas9 for RNA-guided ssRNA binding and/or cleavage (which is denoted RCas9, an RNA targeting Cas9) has been reported (O’Connell et al., 2014), and even more recently, that C2c2 effector functions as an RNA-guided RNA-targeting CRISPR (Abudayyeh et al., 2016).

The double-strand breaks created by Cas9 induce insertion or deletion (indel) mutations in the targeted gene or genome sequence of an organism (Ran et al., 2013), which are repaired by non-homologous end joining (NHEJ) and/or homology directed repair (HDR). NHEJ aligns one to few complementary bases for the re-ligation of two ends, whereas HDR uses longer stretches of sequence homology to repair DNA breaks. Screening, identification and frequency of mutations can be done efficiently and rapidly using NGS rather than the first-generation Sanger sequencing method (Bell et al., 2014).

During the last few years, the CRISPR-Cas based systems have become the method of choice for genome editing by introducing or correcting genetic mutations in a wide variety of biological contexts: cell lines, animals (including humans) and plants (Belhaj et al., 2015), as well as human RNA and DNA viruses (Price et al., 2015; Kennedy and Cullen, 2015). The advantages of the CRISPR-Cas genome editing over the other known genome editing systems are that it is faster, easier to use and applicable to many species, as opposed to being species-specific, as it has been used in organisms recalcitrant to previous attempts at genome engineering. It is also versatile as it can be used to introduce or delete a number of different genes at a time and does not require many manipulating tools.

### Developing Plants Resistant to DNA Geminiviruses

Geminiviruses (family *Geminiviridae)* are circular ssDNA viruses with genomes of 2.3 to slightly above 5 kb, distributed worldwide and transmitted by insects, which cause serious damage to many economically important dicotyledonous and monocotyledonous crops (Moffat, 1999; Shamloul et al., 2001; Mansoor et al., 2003; Vanitharani et al., 2005; Czosnek et al., 2013; Hanley-Bowdoin et al., 2013). They replicate in plant nuclei through dsDNA intermediates that also serve as templates for transcription (Pilartz and Jeske, 1992). Current strategies for controlling geminiviruses vary from conventional breeding of resistant cultivars and insect vectors control to molecular methods based on transgenic plants expressing mutated viral proteins, RNA-mediated interference and others (Pilartz and Jeske, 1992; Vanderschuren et al., 2007; Aragao and Faria, 2009; Reyes et al., 2013; Yang C.-F. et al., 2014; Lapidot et al., 2015). All these strategies have met so far with marginal success.

Very recently, the application of the CRISPR-Cas systems targeting geminiviruses has been shown to enhance resistance to tomato yellow leaf curl virus (TYLCV, genus *Begomovirus*) and bean yellow dwarf virus (BeYDV, genus *Mastrevirus*) in *Nicotiana benthamiana* (Ali et al., 2015; Baltes et al., 2015) and to beet severe curly top virus (BSCTV, genus *Curtovirus*) in *N. benthamiana* and in *Arabidopsis* (Ji et al., 2015).

Ali et al. (2015) engineered sgRNAs targeting coding and non-coding TYLCV sequences, including the conserved non-coding intergenic region (IR) of about 300 nt that can form a stem-loop structure containing the origin of replication and promoter sequences for RNA polymerase II (Czosnek et al., 2013; Mori et al., 2013; Yang X. et al., 2014). SgRNAs targeting the IR were the most efficient in reducing TYLCV DNA titer and attenuating disease symptoms. The sgRNAs-Cas9 system was also successful in targeting simultaneously the three geminiviruses TYLCV, beet curly top virus (genus *Curtovirus*) and merremia mosaic virus (genus *Begomovirus*) when sgRNAs specific for the IR sequence of each virus were used (Ali et al., 2015).

Similarly, Ji et al. (2015) targeted coding and non-coding sequences of BSCTV genome. SgRNA-Cas9 constructs inhibited the virus DNA replication at varying levels. Disease symptoms were also attenuated to different levels, which ranged from severe to mild leaf curly symptoms. Over-expressing sgRNA-Cas9 specifically targeting the viral DNA genome sequences resulted in virus-resistant plants.

It has been shown that two sgRNAs targeting the same BeYDV genome site in infected *N. benthamiana* plants can significantly increase resistance as compared to using only one single sgRNA (Baltes et al., 2015). Moreover, NGS analysis of indels within the viral genome suggested that Cas9 can bind to the viral genome and introduce the dsDNA breaks at the targeted sites, and also indicated that most mutations were 1–2 bp indels.

### Developing Plants Resistant to RNA Viruses

Plant RNA viruses depend on host factors for their replication and infection as their coding capacity is limited (Sanfacon and Jovel, 2007; Nagy and Pogany, 2012; Wang, 2015). Recessive genes, including those coding for the translation initiation factors, confer plant resistance to infection by RNA viruses (Truniger and Aranda, 2009; Sanfaçon, 2015). More specifically, the eukaryotic translation initiation factor eIF4E and its isoform have been identified in cucumber (*Cucumus sativus* L.; Rodríguez-Hernández et al., 2012). In infected plant cells both proteins interact with the small VPg protein of some RNA viruses, such as members of the families *Potyviridae* and *Secoviridae* and of the genus Sobemovirus and alterations in such interactions result in a broad-spectrum resistance to viruses (Sanfaçon, 2015).

It had been shown that engineering mutations in the host plant eIF4E through the use of the Targeting-Induced Local Lesions in Genome (TILLING) strategy could provide resistance against potyviruses in a range of plants (Julio et al., 2015; Gauffier et al., 2016). The CRISPR-Cas9 sgRNA system has now been used to efficiently inactivate these genes and generate virus-resistant plants (Chandrasekaran et al., 2016; Pyott et al., 2016). For example, the targeting of two sites of eIF4E gene in cucumber allowed developing plants resistant to infection by five positive-strand RNA viruses (Chandrasekaran et al., 2016). The targeted gene sites were within the first and third exon sequences. Small indels and small nucleotide polymorphisms (SNPs) were observed in the T1 generation. Homozygous T3 progeny with 20 and four deletions in the eIF4E gene were immune to infection by cucumber vein yellowing virus (family *Potyviridae*, genus *Ipomovirus*), and resistant to papaya ringspot virus-W and zucchini yellow mosaic virus (family *Potyviridae*, genus *Potyvirus*). As expected, the plants remained susceptible to viruses that do not appear to highjack the host eIF4E to complete their cycle, such as cucumber mosaic virus (family *Bromoviridae*, genus *Cucumovirus*) or cucumber green mottle mosaic virus (family, *Virgaviridae*, genus *Tobamovirus*). Pyott et al. (2016) were also successful in engineering complete resistance in *Arabidopsis* to turnip mosaic virus, a potyvirus, using the CRISPR/Cas9 technology targeting the plant elF (iso) 4E gene.

## Conclusion and Prospective

For the last seven years, NGS and bioinformatics have provided rapid and low cost DNA and/or RNA sequencing for plant viruses and viroids. Full genomes or virus- or viroid-specific small RNAs, which cover essentially the whole genome, have been sequenced for discovery of novel pathogens, as well as for pathogen detection and identification, replication, ecology, epidemiology, and pathogen-host interactions. Many novel plant RNA and DNA viruses were successfully discovered but only a couple of novel viroids were revealed. Known viruses and viroids were successfully deteted and identified by NGS. Prior knowledge of virus or viroid sequence was not needed, which has made NGS a universal rapid and accurate method for pathogen discovery and diagnostics. Using NGS in a quarantine program, a novel virus in imported sugarcane was discovered that previously escaped detection by standard detection methods. Similarly, using NGS in a certification program, the causal agent of severe hop stunt disease has been very recently discovered and now the disease is under control. In the very near future NGS may be utilized in plant quarantine and certification programs to significantly increase their capacities and reliabilities. NGS is playing a significant role in connecting plant virology with other related fields of biology such as genome editing. Very recently, CRISPR-Cas9 system has been used in developing plants resistant to five DNA geminiviruses and four RNA potyviruses. Since CRISPR/Cas9 system has the capabilities to work on DNA or RNA sequences, expanding the use of these capabilities in research on DNA/RNA viruses or viroids, in association with NGS, may open widely the door for studying control of diseases caused by these pathogens at the genomic level. These advances are propelled in part by synergies between two powerful technologies: NGS and genome engineering.

## Author Contributions

AH conceived and wrote the manuscript, and RF, TC, and MB revised it and made useful suggestions. AH prepared the final version of the manuscript and all authors approved it.

## Conflict of Interest Statement

The authors declare that the research was conducted in the absence of any commercial or financial relationships that could be construed as a potential conflict of interest. The reviewer VP declared a shared affiliation, though no other collaboration, with one of the authors RF to the handling Editor, who ensured that the process nevertheless met the standards of a fair and objective review.
